# Neurosarcoidosis-like reaction under TNF-α inhibitors: a case report and literature review of a paradoxical immune phenomenon

**DOI:** 10.1007/s10072-026-08955-z

**Published:** 2026-03-16

**Authors:** Gianvito Barbella, Pietro Antenucci, Marta Rosa, Andrea Gozzi, Marina Padroni

**Affiliations:** 1 Unit of Clinical Neurology, Neurosciences and Rehabilitation Department, University of Ferrara, Via Aldo Moro 8, Ferrara Cona, 44124 Italy; 2 Neurology Unit, Interdistrict Department of Neuroscience, Sant’Anna University Hospital, Via Aldo Moro 8, Cona, Ferrara 44124 Italy

**Keywords:** Neurosarcoidosis, Sarcoidosis-like reaction, TNF-α inhibitor, Adalimumab

## Abstract

**Background:**

Neurosarcoidosis (NS) is a rare manifestation of sarcoidosis that oftenrequires long-term immunosuppressive treatment (IST), including tumor necrosis factor-α (TNF-α) inhibitors in refractory cases. Paradoxically, TNF-α blockade has also been associated withsarcoidosis-like reactions (SLRs), granulomatous inflammatory conditions that mimic idiopathicsarcoidosis. Case presentation: We report a case of NS occurring in the context of a TNF-αinhibitor–associated SLR and review previously reported cases during TNF-α inhibitor therapy.

**Discussion:**

A 25- year-old man with HLA-B27–negative ankylosing spondylitis developed anacute central nervous system (CNS) inflammatory syndrome during prolonged adalimumabtherapy. The diagnosis was supported by inflammatory cerebrospinal fluid (CSF) findings,including an elevated CD4+/CD8+ ratio, histologically confirmed pulmonary SLR, and sustainedradiological and neurological remission after adalimumab withdrawal. A review of the literatureidentified only seven reported cases of NS during anti-TNF-α therapy across heterogeneousimmune-mediated inflammatory diseases. Clinical and neuroimaging features were variable,whereas CSF analysis consistently showed inflammatory changes. Exposure duration prior toneurological onset and follow-up strategies were inconsistently reported, and acute IST wasfrequently required because of CNS involvement.

**Conclusions:**

This case expands the clinicalspectrum of anti-TNF-α– associated SLRs and underscores the importance of considering aniatrogenic etiology in paradoxical neuroinflammatory presentations. Recognition of a“neurosarcoidosis-like reaction” may inform long-term therapeutic decisions, including carefulconsideration of TNF-α inhibitor re-exposure and selection of alternative ISTs for the underlyingdisease.

## Introduction

Sarcoidosis-like reactions (SLRs) are systemic granulomatous inflammatory responses that are clinically, radiologically, and histologically indistinguishable from idiopathic sarcoidosis (IS), most commonly occurring during immune-modulating treatments [1], including tumor necrosis factor-α (TNF-α) inhibitors [2]. A key distinguishing feature is remission after drug withdrawal [1]. Central nervous system (CNS) involvement is rarely described in drug-induced SLRs (DISLRs), with most cases presenting with pulmonary, lymphatic, or cutaneous manifestations [1–3]. We report a case of CNS sarcoidosis occurring in the context of a TNF-α inhibitor-induced pulmonary SLR.

## Case presentation

**Fig. 1:**
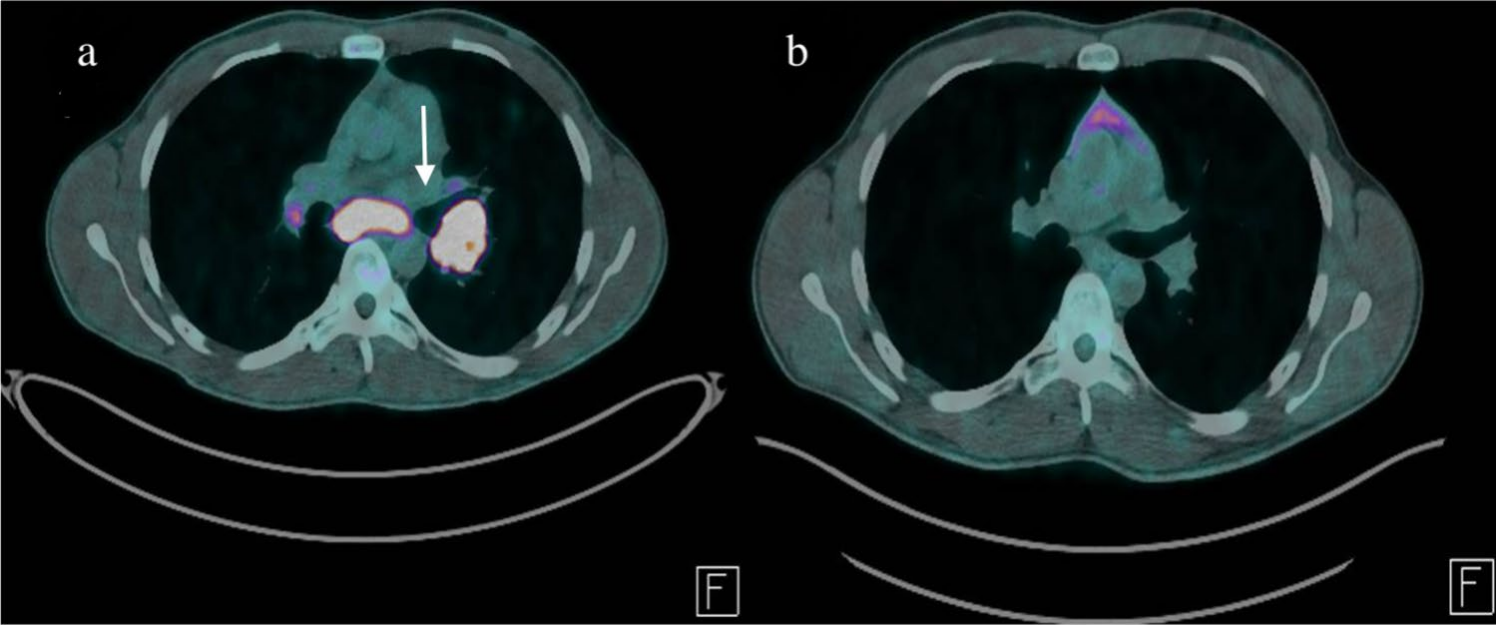
Whole-body 18F-FDG PET scans (**a**) Baseline scan obtained during patient hospitalization, showing multiple hypermetabolic mediastinal lesions (arrow). (**b**) Six-month follow-up scan after TNF-α inhibitor withdrawal, showing marked improvement of abnormal FDG uptake

A 25-year-old man was admitted for speech difficulties with an acute onset 5 h before. He presented a diffuse, severe, pressure-like headache, gradually worsened over the past 2 weeks and poorly responsive to common analgesics. His medical history included active smoking, subclinical hyperthyroidism, and a 10-year history of HLA-B27–negative ankylosing spondylitis (AS) treated with etanercept for 5 years until loss of efficacy, followed by adalimumab (40 mg every 2 weeks) for the subsequent 5 years. He was not taking any other medications at presentation, and his last adalimumab dose was administered 10 days before admission. On neurological examination, the patient was slowed and intermittently drowsy, with a moderate non-fluent aphasia and a mild right-sided hemiparesis. He also complained of photophobia. The remainder of the neurological and general physical examination was unremarkable. No signs or symptoms suggestive of active AS or disease progression were reported. Vital signs showed mild hypotension, while the patient was afebrile. Routine blood tests, including inflammatory markers, as well as toxicological screening, were unremarkable. A direct brain computed tomography (CT) scan, plus CT angiography (CTA) and perfusion imaging, excluded acute ischemic or vascular abnormalities and the electroencephalogram (EEG) showed non-specific focal slowing over the left fronto-parietal region. Intravenous acyclovir was initiated in the suspicion of viral meningoencephalitis after a lumbar puncture had revealed mild cerebrospinal fluid (CSF) lymphocytic pleocytosis (89 cells/µL) with mildly elevated protein levels (109 mg/dL) and mild hypoglycorrhachia (47 mg/dL). The neurological deficits fully resolved within 24 h, although the headache persisted. Brain magnetic resonance imaging (MRI) with gadolinium, performed 36 h after admission, as well as EEG with sleep deprivation were unremarkable. Extensive blood and CSF microbiological investigations, including polymerase chain reaction testing for bacteria, mycobacteria, fungi and common neurotropic viruses, were negative, and acyclovir was therefore discontinued. Serology for cryptococcal, aspergillus, Lyme disease, HIV, syphilis, and hepatitis B and C, was also unremarkable, as well as aerobic and anaerobic bacterial, mycobacterial, and fungal cultures from both blood and CSF. A comprehensive serum and CSF autoimmune work-up, including anti-thyroid antibodies, panel for vasculitis, and an extensive meningoencephalitis panel comprising both surface and intracellular antibodies (including NMDAR, AMPAR1/2, GABA-B receptor, LGI1, CASPR2, GAD, amphiphysin, Ma1/2, and GFAP antibodies), was performed using cell-based assays. All results were negative. Serum and CSF oligoclonal bands were absent and CSF IgG index was within normal limits. Chest CT, prompted by incidental thoracic lymphadenopathy on brain CTA, revealed multiple bilateral pulmonary nodules and mediastinal lymphadenopathy, with increased uptake on whole-body fluorine-18 fluorodeoxyglucose positron emission tomography (18F-FDG PET) (Fig. 1a). Histological examination demonstrated non-caseating granulomatous inflammation, with negative microbiological studies on specimen, consistent with sarcoidosis. Serum angiotensin-converting enzyme (ACE) levels were within normal limits, and interferon-gamma release assay testing was negative. Lactate dehydrogenase, serum protein electrophoresis and CSF cytology were unremarkable. CSF ACE testing was unavailable, while retrospective CSF flow cytometry revealed a CD4⁺ T-cell predominance, with a CD4⁺/CD8⁺ ratio of 5.86, further supporting the diagnosis of NS presenting as aseptic meningoencephalitis. A comprehensive evaluation for other occult extrapulmonary diseases, including skin and ophthalmological examination, electrocardiography and echocardiography, liver function tests, serum and urinary calcium assessment, did not reveal any abnormalities. After a multidisciplinary evaluation, given the suspicion of a TNF-α inhibitor-induced SLR, adalimumab was discontinued and a therapeutic switch to anti-interleukin-17A therapy was planned; oral prednisone (0.5 mg/kg/day) was initiated with a planned taper over 3 months. Following glucocorticoids (GC) initiation, residual headache rapidly improved until resolution. The patient was discharged on day 21, neurologically asymptomatic. A 6-month whole-body 18F-FDG PET, performed while the patient was off both GC and adalimumab, showed complete resolution of previously reported lesions, consistent with SLR diagnosis (Fig. 1b). Twelve months after discharge, secukinumab was initiated as AS immunosuppressive therapy (IST). During a 2-year neurological follow-up, the patient reported no recurrence of neurological or systemic symptoms.

## Discussion and literature review

Neurosarcoidosis (NS), defined as central or peripheral nervous system involvement in sarcoidosis, represents a rare manifestation of the disease, reported in approximately 5–10% of patients with IS, and is the initial presentation in about 10% of cases. The most common non-neurological involvement is pulmonary disease and thoracic lymphadenopathy [4].

Neurological manifestations are heterogeneous and may involve cranial nerves, meninges, brain parenchyma, spinal cord, or, less commonly, peripheral nerves. Notably, symptoms are frequently reported even in the absence of overt granulomatous inflammation [4]. Cranial neuropathies, particularly involving the facial and optic nerves, represent the most common presentation, followed by headaches, sensory disturbances, pyramidal weakness, seizures, and cognitive changes [5]. Symptomatic meningeal involvement is observed in 10–20% of NS patients, presenting as subacute or chronic leptomeningitis, pachymeningitis, or dural-based mass lesions, often associated with cranial neuropathies due to predilection for the skull base [4]. Leptomeningeal involvement can be focal or diffuse and may be more disabling than pachymeningeal forms, frequently requiring early therapeutic escalation [6]. Parenchymal involvement occurs in 30–50% of NS cases [5] and may arise secondary to meningeal spread [7]. In such cases, patients may develop a subacute meningoencephalitic syndrome characterized by headache, fluctuating confusion, behavioural or cognitive changes, seizures, or focal neurological deficits [4–7]. Subcortical encephalopathy, including progressive cognitive impairment, has also been described [7]. Brain MRI with gadolinium is the imaging modality of choice for suspected NS and plays a central role in diagnosis and monitoring of treatment response [6, 7]. The most commonly observed abnormalities include nonspecific periventricular white matter lesions and enhancing intraparenchymal lesions without central necrosis, which may mimic demyelinating disease but typically persist over time [8]. Leptomeningeal involvement often appears as diffuse or nodular contrast enhancement, particularly at the skull base [4, 8]. Nevertheless, MRI findings are not pathognomonic, and differentiation from inflammatory, infectious, or neoplastic mimics may be challenging [4, 9]. Moreover, MRI may be normal or non-diagnostic in a subset of patients [6, 10–12]. Both prospective and retrospective cohorts have documented clinically compatible CNS involvement despite unremarkable imaging [10–12]. Notably, Sambon et al. reported patients with meningeal involvement despite normal brain MRI [12]. Collectively, these observations indicate that MRI negativity does not exclude active NS and highlight the complementary role of CSF examination [6, 10–12], which should be performed whenever feasible to exclude infectious and neoplastic mimics. Although findings are not specific, CSF inflammatory abnormalities are common, particularly in meningeal involvement. Typical features include lymphocytic pleocytosis and elevated protein levels, while CSF glucose is usually normal but may be mildly reduced, especially in patients with meningeal or parenchymal disease [4, 5]. Oligoclonal bands are usually absent or matched in serum, and biomarkers such as CSF ACE have limited diagnostic value. Elevated CSF CD4⁺/CD8⁺ ratio has been associated with active disease [5]. Serum testing is primarily useful to assess systemic involvement and exclude alternative etiologies, as no circulating marker, including acute-phase reactants and ACE, is disease-specific. Accordingly, diagnosis relies on integration of clinical phenotype, imaging, CSF findings, systemic evaluation, and, when feasible, histopathology [7]. Nervous system biopsy remains the diagnostic gold standard but is often impractical due to procedural risk [9]. The differential diagnosis is broad and includes granulomatous infections (particularly mycobacterial and fungal), lymphoproliferative disorders, demyelinating disease, and other inflammatory conditions. In leptomeningeal presentations, differentiation from infectious meningitides, carcinomatous meningitis, and autoimmune syndromes such as GFAP antibody-associated meningoencephalitis is critical [7]. Given the absence of pathognomonic radiological or laboratory markers, diagnostic criteria for NS have evolved toward pragmatic approaches that allow diagnosis even without nervous tissue biopsy, based on evidence of granulomatous involvement of the nervous system on MRI, CSF, and/or neurophysiological studies in patients with systemic sarcoidosis, after rigorous exclusion of alternative diagnoses [13, 14].

Unlike pulmonary sarcoidosis, idiopathic NS rarely undergoes spontaneous remission, except for facial nerve palsy, and most patients require early treatment [4, 6]. Some authors propose stratifying NS according to severity to guide therapeutic intensity: mild forms (e.g., isolated facial nerve palsy), moderate forms (e.g., isolated dural meningeal involvement, other cranial neuropathies, or peripheral neuropathy), and severe forms (e.g., leptomeningeal disease, intraparenchymal brain lesions, or spinal cord involvement) [4]. In clinical practice, treatment decisions must integrate disease severity and extent, functional impairment, comorbidities, and likelihood of treatment response, with active inflammatory features on CSF analysis and contrast-enhancing lesions on MRI suggesting a potentially better response to immunotherapy and often warranting a more aggressive approach [4]. Patients with isolated facial nerve palsy typically respond well to GC and have a relatively low relapse risk; in such cases, a single course of GC without steroid-sparing agents may be sufficient [4, 6]. Conversely, moderate-to-severe disease is less likely to remit spontaneously and frequently relapses during steroid tapering. Accordingly, early introduction of a steroid-sparing IST is generally recommended. Methotrexate and mycophenolate mofetil have demonstrated benefit in both systemic and neurological disease, whereas data supporting the use of azathioprine and cyclophosphamide remain limited [4, 6]. Among second-line ISTs, TNF-α inhibition with infliximab has the strongest evidence in refractory NS, including severe leptomeningeal and pachymeningeal disease of the brain and spinal cord [6], whereas adalimumab is the most frequently used alternative TNF-α inhibitor, although supporting data are limited [4].

However, anti-TNF-α agents have also been associated with the paradoxical development of NS, although this phenomenon is exceedingly rare [15]. To our literature, only seven cases of NS occurring during anti-TNF-α therapy, consistent with the consensus diagnostic criteria proposed by Stern et al. [13], have been reported [16–22]; only one involved a patient with AS [17]. Patients were affected by various rheumatologic diseases, including rheumatoid arthritis, psoriatic arthritis, Crohn’s disease, and HLA-B27-positive AS. Among the offending drugs, infliximab, etanercept and adalimumab were reported. Clinical presentations were heterogeneous, as well as neuroimaging findings, while CSF analysis consistently showed an inflammatory pattern with lymphocytic pleocytosis and elevated protein levels [16–22]. Notably, Sturfelt et al. described a case with neurological features suggestive of aseptic meningoencephalitis, in which neuroimaging findings were unremarkable [16], similarly to our patient. Importantly, in all identified cases, neurological involvement was the presenting feature, driving the diagnostic evaluation and leading to the recognition of sarcoidosis. Isolated neurological involvement was occasionally described. The duration of TNF-α inhibition before neurological onset and follow-up timing were inconsistently reported across cases; when not explicitly stated, only minimum exposure intervals or qualitative follow-up descriptors could be retained [16–22]. The main characteristics of these cases are summarized in Table 1.

**Table 1:** Literature overview of neurosarcoidosis cases during anti-TNF-α therapy

Authors, year	Underlying disease (age/sex)	TNF-α inhibitor (exposure duration)	Neurological presentation	Neuroimaging features	CSF features	Systemic sarcoidosis evidence	Treatment and outcome (follow-up)
Sturfelt et al., [16]	RA (41/F)	IFX (69 mo)	Headache, diplopia, bilateral papilledema, left ocular motor palsy	Brain CT, brain and spinal cord MRI, MRA: normal; signs of raised ICP on TCD	Lymphocytic pleocytosis (49/µL), elevated proteins (78 mg/dL), increased ACE	Bilateral granulomatous iridocyclitis and retinal periphlebitis, increased mediastinal lymph nodes and parotid glands activity on scintigraphy	VPS: headache resolution, slow ophtalmologic improvement; oral HDS + MTX: resolution of ocular findings (~ 6 mo)
Mao-Draayer et al., [17]	HLA-B27 positive AS (36/M)	ADA (36 mo)	New-onset seizures	Brain MRI: leptomeningeal thickening and enhancement, multifocal T2-hyperintensities	Mild lym-phocytic pleocytosis and elevated proteins (details not reported)	None reported (sarcoidosis evidence on brain biopsy)	ADA withdrawal + IV steroid pulses + MTX: remission (NR)
Durel et al., [18]	RA (40/F)	ETN (~ 96 mo)	Bilateral CN VII palsy, anosmia, papilledema	Brain MRI: bilateral CN (V, VII, VIII) enhancement	Lymphocytic pleocytosis (details not reported), elevated proteins (62 mg/dL)	Hilar/mediastinal lymphadenopathy on CT; hilar and salivary gland uptake on PET	ETN withdrawal + IV steroid pulses + MTX: partial clinical improvement, radiological remission (12 mo)
Berrios et al., [19]	RA (33/F)	ETN (≥ 24 mo)	Altered mental status, fever, headache	Brain MRI: leptomeningeal enhancement, diffuse sulcal T2-FLAIR hyperintensities	Lymphocytic pleocytosis (28/µL), elevated proteins (48 mg/dL)	Mediastinal, abdominal and inguinal lymphadenopathy and hepatosplenomegaly; non-caseating granulomas on lymph node biopsy	ETN withdrawal + HDS + MTX + IFX: clinical and radiological improvement (24 mo)
Hunter et al., [20]	RA (61/F)	ETN (NR)	Multiple CN (V, VII, VIII) deficits	Brain MRI: right V and VII enhancement	Pleocytosis (45 cells/µL), elevated proteins (47 mg/dL)	None detected	ETN withdrawal + HDS: clinical (3 mo) and neuroimaging improvement (4 mo)
Nnodum et al., [21]	PsA (63/M)	ADA (≥ 24 mo)	Fever, left lower extremity weakness, unsteady gait, urinary retention	Brain MRI: multifocal T2-FLAIR hyperintensities	Lymphocytic pleocytosis (101/µL), elevated proteins (55 mg/dL)	Pulmonary nodules, mediastinal and hilar lymphadenopathy, multiple liver lesions	ADA withdrawal: neurological remission (NR)
Tun et al., [22]	CD (32/M)	ADA (NR)	Headache, confusion, new-onset seizure	Brain MRI: multifocal white-matter lesions	1 st LP: pleocytosis (18/µL), raised proteins (859 mg/L); 2nd LP: lymphocytic pleocytosis (110/µL), elevated proteins (1064 mg/L), elevated ACE, OCBs present	Bilateral hilar and mediastinal lymphadenopathy, with non-caseating granulomas on biopsy	ADA withdrawal + IV steroid pulses + chronic steroids: neurologicalRemission (NR)

*ACE*, Angiotensin-converting enzyme; *ADA*, Adalimumab; *AS*, Ankylosing spondylitis; *CD*, Crohn’s disease; *CN*, Cranial nerves; *CSF*, Cerebrospinal fluid; *CT*, Computed tomography; *ETN*, Etanercept; *F*, Female; *FLAIR*, Fluid-attenuated inversion recovery; *HDS*, High-dose steroids; *ICP*, Intracranial pressure; *IFX*, Infliximab; *IV*, Intravenous; *LP*, Lumbar puncture; *M*, Male; *mo*, Months; *MRA*, Magnetic resonance angiography; *MRI*, Magnetic resonance imaging; *MTX*, Methotrexate; *NR*, Not reported; *OCBs*, Oligoclonal bands; *PET*, Positron emission tomography; *PsA*, Psoriatic arthritis; *RA*, Rheumatoid arthritis; *TCD*, Transcranial doppler; *VPS*, Ventriculoperitoneal shunt

Although current diagnostic criteria for DISLRs emphasize clinical improvement after drug discontinuation rather than response to GC or other immunosuppressive drugs [3], most authors interpreted the neurological manifestations as related to anti-TNF-α exposure and consistent with a sarcoid-like granulomatous process. This interpretation was often made despite the absence of a uniform diagnostic framework, with heterogeneous terms such as “paradoxical neurosarcoidosis”, “CNS sarcoidosis”, or simply “neurosarcoidosis” being used across the reported cases. Concurrently, acute treatment with GC, methotrexate, or alternative TNF-α inhibitors was frequently reported, and in several cases the administration or discontinuation of these therapies at follow-up was not clearly specified [16–22]. Of note, only one case described neurological remission following TNF-α inhibitor withdrawal alone, without additional pharmacological treatment [21]. At the same time, current consensus statements acknowledge that systemic GC are often required in the acute management of severe or potentially organ-threatening manifestations of DISLRs, as is the case for CNS, making a period of observation after drug withdrawal alone frequently impractical; in this context, sustained neurological remission after complete treatment withdrawal appears as the key distinguishing feature [3]. As a result, distinguishing idiopathic NS from a DISLR can be clinically challenging; however, this distinction carries substantial diagnostic and therapeutic implications. The diagnosis of DISLR is inherently longitudinal, requiring integration of temporal association with drug exposure, comprehensive systemic evaluation, and structured clinical-radiological follow-up to document sustained remission after drug withdrawal [1, 3]. As discussed above, TNF-α inhibition is particularly beneficial in idiopathic NS with severe meningeal disease [6]. Conversely, when CNS granulomatous inflammation develops in the setting of anti-TNF-α exposure, continuation or re-initiation of the same drug class may be inappropriate [3]. Early recognition of a DISLR is therefore critical, as it may prevent misclassification as idiopathic NS, avoid inappropriate therapeutic escalation, and reduce the risk of re-exposure to a potential immunological trigger. Moreover, accurate identification of a DISLR may allow transition to alternative non–TNF-α therapies once clinical stability is achieved, thereby limiting cumulative immunosuppressive burden while ensuring appropriate control of the underlying disease.

Monoclonal TNF-α inhibitors, such as adalimumab, likely increase the peripheral Th1/Th2 ratio, leading to enhanced IFN-γ production, macrophage activation, and granuloma formation; IFN-γ, in turn, reinforces Th1 differentiation, promoting a self-amplifying inflammatory loop [23–25]. Beyond this Th1-driven mechanism, sarcoidosis is also characterized by a state of systemic immune dysregulation, in which regulatory CD4⁺CD25⁺FoxP3⁺ T cells, at least in part dependent on TNF-α signalling, contribute to peripheral immune anergy [26]. TNF-α blockade may therefore paradoxically impair immune regulatory pathways, favouring a compensatory shift toward IL-23/Th17 pathway activation, with increased IL-17-mediated inflammation [27]. The convergence of Th1 and Th17 loops may provide a biologically plausible framework for paradoxical SLRs, including CNS involvement [26, 27].

In our patient, the development of an inflammatory syndrome during prolonged TNF-α inhibitor therapy, together with the absence of relapses after drug withdrawal and throughout a sustained off-drug period, supported the diagnosis of a systemic adalimumab-induced SLR. Notably, CSF inflammatory findings, including an elevated CD4⁺/CD8⁺ ratio (> 5), associated with active NS [5, 28], and mild hypoglycorrhachia, a feature occasionally suggestive of sarcoid meningitis [4, 5], pointed toward a shared pathophysiological mechanism for the neurological involvement, consistent with CNS manifestation of the DISLR. To our knowledge, this is the first reported case of NS occurring during TNF-α inhibitor therapy in a patient with HLA-B27–negative AS. Beyond expanding the clinical spectrum of SLRs to include CNS involvement, this case underscores the importance of considering this etiology in patients receiving anti-TNF-α agents who present with paradoxical neuroinflammatory events, even in the presence of unremarkable neuroimaging. While acute management may overlap with idiopathic NS, early recognition of a DISLR in this context may guide long-term therapeutic decisions, including avoidance of TNF-α inhibitors in favor of alternative ISTs.
